# Effects of Per- and Polyfluoroalkylated Substances on Female Reproduction

**DOI:** 10.3390/toxics12070455

**Published:** 2024-06-25

**Authors:** María Estefanía González-Alvarez, Collins Antwi-Boasiako, Aileen F. Keating

**Affiliations:** Department of Animal Science, Iowa State University, Ames, IA 50011, USA

**Keywords:** per- and polyfluoroalkylated substances, ovary, endocrine, cyclicity

## Abstract

Per- and poly-fluoroalkylated substances (PFAS) are a large group of chemicals that persist both in the environment and in the body. Legacy PFAS, e.g., perfluorooctanoic acid and perfluorooctane sulfonic acid, are implicated as endocrine disruptors and reproductive and developmental toxicants in epidemiological and animal model studies. This review describes female reproductive outcomes of reported studies and includes where associative relationships between PFAS exposures and female reproductive outcomes have been observed as well as where those are absent. In animal models, studies in which PFAS are documented to cause toxicity and where effects are lacking are described. Discrepancies exist in both human and animal studies and are likely attributable to human geographical contamination, developmental status, duration of exposure, and PFAS chemical identity. Similarly, in animal investigations, the model used, exposure paradigm, and developmental status of the female are important and vary widely in documented studies. Taken together, support for PFAS as reproductive and developmental toxicants exists, although the disparity in study conditions and human exposures contribute to the variation in effects noted.

## 1. Introduction

Per- and poly-fluoroalkylated substances (PFAS) are a family of more than 4000 chemicals [1], from which more than 600 are currently being commercially used [2]. The Organisation for Economic Co-operation and Development (OECD) define PFAS as “fluorinated substances that contain at least one fully fluorinated methyl or methylene carbon atom (without any H/Cl/Br/I atom attached to it)” [3]. PFAS are characterized by having very strong bonds between carbon and fluorine atoms [4,5,6], which gives them thermal and chemical stability [7] and causes them to be persistent in the environment [6]. Since PFAS chemicals have both hydrophobic and lipophobic tails and a polar hydrophilic head [5,8,9], they repel water and oil and have thus been widely used in commercial and industrial products since the 1940s [7,10]. Their carbon length and functional groups vary [1], and they can be classified as long and short chains [11]. Long chain PFAS are defined as perfluoroalkyl carboxylic acids (PFCAs) with eight carbons and greater and perfluoroalkane sulfonates (PFSAs) with six carbons and greater [11]. Short-chain PFAS are defined as PFCA with seven or fewer carbons and PFSA with five or fewer carbons. This difference in the number of carbons is because PFSA compounds tend to bioaccumulate more than PFCAs with the same number of carbon atoms [11]. Specific structures of individual PFAS chemicals are accessible through the US EPA CompTox Chemicals repository.

Long-chain PFAS include legacy chemicals such as perfluorooctanoic acid (PFOA) and perfluooroctane sulfonic acid (PFOS), perfluorononanoic acid (PFNA), perfluorodecanoic acid (PFDA), and perfluorohexane sulfonic acid (PFHxS) [11,12]. Short-chain PFAS include perfluorobutanoic acid (PFBA), perfluorohexanoic acid (PFHxA), and perfluorobutanesulfonic acid (PFBS) [13]. In addition, hexafluoropropylene oxide (HFPO) dimer acid and its ammonium salts, better known as GenX and 3H-4,8-dioxanonanoate (ADONA), are also short-chain PFAS that were introduced to replace PFOA, while chlorinated polyfluoroalkyl ether sulfonate (F53B) was introduced to replace PFOS [14]. In the 2000s, a voluntary PFOS phase-out was initiated, followed by the United States Environmental Protection Agency (USEPA) PFOA Stewardship Program to eliminate PFAS emissions and products [15,16]. With the phase-out of long-chain PFAS, the use of short-chain PFAS was extended to replace PFAS chemicals; however, these PFAS are less regulated, and despite having shorter half-lives of elimination in organisms, they are as persistent in the environment as long-chain PFAS and are also extensively distributed [13,17].

Due to their physicochemical properties, PFAS are used in a myriad of consumer products, including fabric coatings, non-stick cookware, fire-fighting foams, food packaging, and personal care products [18,19,20]. Human and animal exposure to PFAS is through ingestion, inhalation, and dermal exposures [18,21], and PFAS are present in the blood of the majority of humans living in industrialized countries [22,23,24]. In general, PFAS bind to albumin [17,25] and accumulate in the blood, liver, kidneys, testicles, brain [9,26,27], and ovaries [28] but do not tend to accumulate in adipose tissue [8,21,27]. However, one study found that PFAS were present in the adipose tissue of pigs, attributing this difference to the protonated or deprotonated form of PFAS [29]. These compounds are typically not metabolized *in vivo*, hence their long half-lives [18,28,30,31]. Elimination half-lives vary between species and chemical types [17,30]. PFAS can be eliminated through urine, feces, and bile [18,28,30] and can also be excreted through breast milk [18] and menstrual fluid [32,33]. In general, the half-life of elimination decreases in PFAS with a shorter carbon chain, and half-lives of elimination in humans and other animals are summarized in Table 1 [28,29,34,35,36,37,38,39,40,41,42,43,44,45,46,47,48,49,50,51,52,53,54,55,56,57,58,59,60]. Furthermore, there are biological sex differences in the half-life of PFAS elimination. For example, in female rats, the half-life of PFOA is around 2–4 h, while in male rats, it is 4–9 days [28,41]. This difference is explained by differences in secretory mechanisms in the kidney between female and male rats [28]. Nevertheless, this is not the case for all species; for example, in non-human primates, PFOA has an apparent longer half-life of elimination in females (32 d) compared to males (21 d) [55]. In mice, the half-life of PFOA in females is 15 days, and in males, 21 days [52], potentially translating to their serving as an appropriate model to study potential human toxic effects [21]. Another important detail is the difference in the half-life of elimination between some species [61]; for example, the half-life of elimination of PFOS, PFOA, and PFHxS in rodents is much shorter compared to humans [28,34,35,41,42,45,47,48,52]; however, in pigs, PFAS have longer time lengths for elimination compared to other species [29]. While elimination half-lives for long-chain PFAS are reported, studies to determine the half-life of elimination of the newer PFAS and their replacements remain necessary. 

PFAS can be easily absorbed after oral ingestion [12,62] and are detected in drinking water [21,63,64], animal food products [1,65,66], and bodily fluids [67,68]. As noted above, PFAS have been detected in the serum of most of the U.S. population [67,68,69,70,71] and are reportedly higher in children [72]. The average human exposure to PFOA and PFOS in 2015–2016 was 1.56 and 4.72 ng/mL [73,74], while high PFOA and PFOS exposure levels ranged from 47–128 and 30–219 ng/kg/day, respectively [75]. However, serum concentrations of short-chain PFAS have trended towards being increased [32]. In 2022, the USEPA established interim drinking water health advisories for PFOA (0.004 ppt), PFOS (0.02 ppt), GenX chemicals (10 ppt), and PFBS (2000 ppt) [76]. In addition, the USEPA has proposed a National Primary Drinking Water Regulation for six PFAS, with enforceable maximum contaminant levels (MCLs) set at 4 ppt for PFOA and PFOS individually and 10 ppt for PFNA, PFHxS, and HFPO-DA (GenX chemicals) [77]. Under the Stockholm Convention on Persistent Organic Pollutants, PFOS were listed in 2009 in Annex B, which restricts the production and use of the chemicals listed [78]. Furthermore, PFOA and PFHxS were recently listed as Annex A chemicals [78], and long-chain PFCAs are being reviewed to determine their annex listing [78]. In the European Union, the Scientific Panel on Contaminants in the Food Chain (CONTAM) of the European Food Safety Authority (EFSA) established the tolerable daily intake (TDI) for PFOS and PFOA as 150 ng/kg and 1500 ng/kg body weight per day, respectively [5]. The TDI for production animals, however, remains unclear. PFAS are reported to adversely affect health in humans and animal models, including liver and kidney disease, cancer, lipid and insulin alterations, changes in the immune system, alterations in the thyroid function, endocrine disruption, and reproductive and developmental toxicity [5,18,61]. This review will focus on PFAS’ effects on female reproduction with a description of available human and animal studies.

## 2. Female Reproduction

The female reproductive system is comprised of the oviducts, uterus, cervix, vagina, ovaries, and external genitalia [79,80]. The ovary is a dense structure in the pelvic cavity near the lateral walls [80], composed of somatic and germ cells [81]. The two primary functions of the ovary are (1) production and release of oocytes through the processes of oogenesis and folliculogenesis and (2) production and secretion of hormones (17β-estradiol (E_2_) and progesterone (P_4_)), which are essential for the proper functioning of the female reproductive system [79,82,83] and female general health [84].

The process of folliculogenesis describes how immature oocyte-containing follicles develop and mature to be ovulated or die by atresia [79]. Primordial follicles are the most immature follicle stage present in the ovary [79] and are comprised of the oocyte surrounded by a single layer of flattened or squamous granulosa cells that are surrounded by a basal lamina [79,81,85]. Local ovarian factors activate the primordial follicles to develop into primary follicles, upon which an increase in oocyte size is observed [80,85]. The flattened granulosa cells surrounding the oocyte become cuboidal granulosa cells [79,80,81,85]. The zona pellucida, a non-cellular layer, also appears at this stage of follicular development, surrounding the oocyte, and it is preserved until ovulation [81,85]. Granulosa cells are essential for the nutrition and support of the oocyte, synthetizing factors that are trafficked to the oocyte by diffusion through the zona pellucida [86]. The secondary follicles develop from primary follicles and are referred to as pre-antral follicles, containing multiple layers of granulosa and theca cells [81]. The newly recruited theca cell layer contains blood vessels and nerves and supports the follicle [81]. Secondary follicles also have a network of gap junctions consisting of connexins, proteins necessary for follicular development, and connect to adjacent cells, allowing nutrients, small metabolites, and second messengers to pass from cell to cell [85]. Only some follicles proceed to the next developmental stage when they are known as tertiary or antral follicles, which are dependent upon follicle-stimulating hormone (FSH) [81,85].

Follicular fluid (FF) is formed from filtered blood circulating in the thecal capillaries [87] and from granulosa cells. This fluid accumulates and separates the inner and outer layers of the follicle to form an antral cavity [80,81,85]. Most of these follicles will undergo atresia; the remaining will grow to the preovulatory stage [85]. Preovulatory follicles produce E_2_, which rises in concentration, resulting in positive feedback on the hypothalamus and pituitary to precipitate the luteinizing hormone (LH) surge, critical for ovulation of the oocyte [81,85]. The LH surge decreases E_2_ production and increases P_4_ secretion [81]. The remaining granulosa and theca cells luteinize to form the corpus luteum (CL), producing P_4_ to prepare the uterus in case pregnancy occurs [81,85]. If pregnancy does not occur, the CL will degenerate, becoming a corpus albicans, marking the end of the ovarian cycle [80].

The entire ovarian reserve of oocytes is produced during fetal development [88,89]. A human female has 14 million oocytes at 20 weeks of gestation, but this number will decline during the female’s life [81,89,90]. At the time of birth, the ovary contains ~1–2 million oocytes; at puberty, this number drops to ~300,000, and at menopause, the ovary has <100 oocytes [81,89,91,92,93,94,95]. Of these, only 400–500 oocytes are ovulated during a woman’s lifetime [81,93].

As noted, steroid hormones are produced in and act on the ovary, similar to other tissues [81]. In the ovary, E_2_ and P_4_ are synthesized from circulating cholesterol [81,96] through the two-cell two-step theory of steroidogenesis. Cholesterol is converted via a series of enzymatic reactions to form testosterone in the theca cell and aromatized to E_2_ via the action of cytochrome P450 isoform 19A1 in the granulosa cell [97]. Thus, the ovary is a dynamic organ vital for the production of the female gamete and critical to proper endocrine balance in females.

## 3. Ovarian Toxicity

In the United States (US), 6.1 million women (10%) aged 15–44 years have difficulty conceiving [98]. As mentioned previously, women are born with a finite number of oocytes and anything that disrupts reproductive health can lead to temporary or permanent infertility [99]. The average age at the onset of menopause in the US is 51 years, and it results from the cessation of ovarian cyclicity due to the depletion of the ovarian follicular pool [100]. Menopause onset before 40 years is referred to as premature ovarian failure (POF) and can be induced by an increase in levels of gonadotropins or hypoestrogenism leading to a depletion of the ovarian follicular pool [101] and alterations in the hypothalamic–pituitary–ovarian axis [102].

Environmental, occupational, medicinal, or xenoestrogenic chemicals can also cause adverse effects on the female reproductive system [83,103,104,105,106,107,108,109,110,111]. Chemicals that affect ovarian function are known as ovotoxicants and can target different stages of follicular development [88,106,112], leading to harmful effects on follicle development, decreased oocyte quality and ovulation, disruption of the estrous cycle, and altered hormonal production [106,108,113,114,115]. Depletion of primordial follicles by ovotoxicants can cause POF and permanent infertility due to the loss of the follicle pool that is irreplaceable [88,99,100,112,116,117]. Damage to growing or antral follicles can disrupt the menstrual cycle by altering ovarian steroid production and impairing ovulation, but the damage is temporary because these follicles can be replaced from the primordial follicle pool [88,99,108,116]. Many factors can influence ovarian toxicity, including the concentration and duration of chemical exposure, as well as the age at which the exposure occurs [112]. Thus, ovotoxicity is a broad term comprising a range of phenotypic outcomes of toxicant exposure.

## 4. Effect of PFAS on Human Female Reproduction as Determined by Epidemiological Studies

Several studies have associated exposure to PFAS with adverse effects on female reproduction. However, most have evaluated the effects of long-chain or legacy PFAS, and inconsistencies, as noted throughout this review, exist amongst these associative findings. Epidemiological studies have linked PFAS exposure with alterations in reproductive hormone levels, menarche and menopause onset, menstrual cycle length, endometriosis, polycystic ovary syndrome (PCOS), and impaired fertility.

### 4.1. Endocrine Disruption

PFAS are reported as endocrine disruptors [9], and exposure to PFAS is postulated to alter the regulation of the hypothalamus–pituitary–ovarian axis [118]. In girls between 6–9 years old, high PFOS serum concentrations are associated with lower total testosterone and insulin-like growth factor-1 (IGF-1) levels [119]. In addition, levels of PFNA are also inversely associated with IGF-1 levels [119]. In Taiwanese girls aged between 12–17 years, there were no links determined between PFAS serum concentration and serum E_2_, FSH, and LH, with the exception of perfluoroundecanoic acid (PFUnA), which was correlated with decreased FSH levels, and both PFOS and perfluorododecanoic acid (PFDoA) were inversely associated with serum testosterone levels [120,121]. In contrast, testosterone concentrations were higher in 15-year-old girls who were prenatally exposed to PFOS, PFOA, and PFHxS [122]. Interestingly, another study in 20-year-old women did not link prenatal exposure to PFOS and PFOA with levels of E_2_, testosterone, FSH, or LH [123]. However, inverse associations were reported for E_2_ and P_4_ levels and PFOS concentrations in women, but a lack of any link was reported for the other PFAS, including PFOA [33,124]. In women aged between 20–45 years, serum testosterone concentrations were positively associated with exposure to PFOA, PFHxS, and PFNA [125]. In midlife women during the menopausal transition, PFOA and PFOS exposure showed a positive connection with FSH levels, while PFNA and PFOA had an inverse relationship with E_2_ in circulation [126]. The potential for PFAS exposure to affect reproductive hormone levels is concerning and could contribute to negative effects on female reproduction and general health.

### 4.2. Puberty and Menopause Onset

Exposure to PFAS has also been associated with differences in cycle length, puberty initiation, and menopause onset, alterations which may affect the proper functioning of the reproductive system and might lead to infertility.

### 4.3. Puberty and Menopause

Exposure to PFAS has been correlated with altered timing of puberty and menopause onset, which are both important for female reproductive and general health. Not many epidemiological studies have evaluated the timing of puberty onset and correlations with PFAS exposure, with inconsistent results in those reported. Three studies have illustrated links between high levels of PFOA and PFOS with delayed puberty in girls [123,127,128]. Early puberty onset has been correlated with exposure to PFOS, PFHxS, PFHps, PFNA, and PFDA [129]. Similarly, there are links reported between higher PFAS serum levels and early menopause onset, albeit with discrepancies also existing [33,130]. Interestingly, PFAS serum concentrations in premenopausal women are lower than in postmenopausal women, who may bioaccumulate PFAS once they no longer menstruate [130,131,132,133]. Reverse causation has been proposed as a possible explanation for differences in time to pregnancy, early menopause, and PFAS levels in women [33,130,134]. Since PFAS may be excreted through endometrial lining shedding during menstruation, women who no longer menstruate may have higher levels of PFAS; the same would apply to women that have longer interpregnancy intervals, who may have experienced a greater number of menstrual cycles to eliminate more PFAS [33,130,134].

### 4.4. Menstrual Cyclicity

PFAS exposures have been shown to cause irregular menstrual cycles [118]. Increased odds of irregular menstrual cycles have been associated with exposure to PFOA [128]. Higher PFOS concentrations have also been associated with irregular menstrual cyclicity, but there were no associations with other PFAS, including PFOA, PFHxS, and PFNA [125]. Similarly, another study did not note a correlation between exposure to PFOS and PFOA and menstrual cycle length [123]. Different PFAS may change menstrual cycle length in both directions; for example, exposure to PFOA decreases menstrual cycle length, but conversely, exposure to perfluorodecanoate (PFDeA) increases the duration [135]. In women from Greenland, Poland, and Ukraine, higher levels of PFOA were linked with longer menstrual cycles [136], while higher PFOS levels were less firmly related to irregular menstrual cycles [136]. Positive associations between PFOA, PFOS, PFNA, and PFHxS and self-reported longer menstrual cycles were reported in Chinese women [137]. After adjusting for confounders, self-reported irregular menstrual cycles were generally not associated with PFAS serum concentrations in the Norwegian Mother and Child Cohort [138]. However, shorter cycles were associated with lower serum concentrations of perfluoroheptane sulfonate (PFHpS) and PFOS in parous women [138]. In addition, in women using oral contraceptives, longer menstrual cycles were associated with higher PFNA and PFUnA concentrations [138]. Alterations in the length of the menstrual cycle and cyclicity are important since they can lead to problems with normal endocrine homeostasis and fertility.

### 4.5. Fecundity Indices

In women aged 35–44 years, there was no association between fecundability ratios and anti-Müllerian hormone (AMH), which is used clinically as a marker of ovarian reserve [139,140] and PFOA, PFOS, PFNA, and PFHxS serum levels; however, women with higher serum PFAS concentrations had longer mean cycle lengths and were less likely to achieve pregnancy by the cessation of the study [141]. In agreement with these findings, a relationship between PFOA, PFOS, PFNA, and PFHxS prenatal exposure was not reported with AMH levels in adolescents (14–16 years) and young adult (20 years) female offspring [123,142]. In addition, higher concentrations of PFOS, PFOA, PFHxS, and perfluorooctane sulfonamide (PFOSA) were associated with a longer time to pregnancy [118,143,144]. Serum PFOS and PFOA were linked to reduced fecundity [145], while PFOA and PFNA were associated with a lower probability of pregnancy [135]. In contrast, a lack of association between PFAS (PFOS, PFOA, PFHxS, PFNA, PFDA, PFOSA, N-methyl-perfluorooctanoic sulfonamidoacetate (MeFOSAA), and N-ethyl-perfluorooctane-sulfonamidoacetate (EtFOSAA)) concentrations and time to pregnancy has also been noted [146] and limited support for an association between time to pregnancy and plasma concentrations of PFOSA was noted in primiparous women in the Norwegian Mother and Child Cohort Study [147]. In women from Greenland, Poland, and Ukraine, consistent findings between PFOA, PFOS, and PFHxS levels and infertility were absent [148]. Nonetheless, high levels of PFNA were associated with a longer time to pregnancy and odds ratio for infertility in women from Greenland, but these associations did not repeat after conducting a sensitivity analysis of primiparous women [148]. In general, there is support for PFAS exposure being associated with longer time to pregnancy and fecundity, though a discrepancy certainly exists in the literature.

### 4.6. PFAS in Follicular Fluid

**Figure 1:**
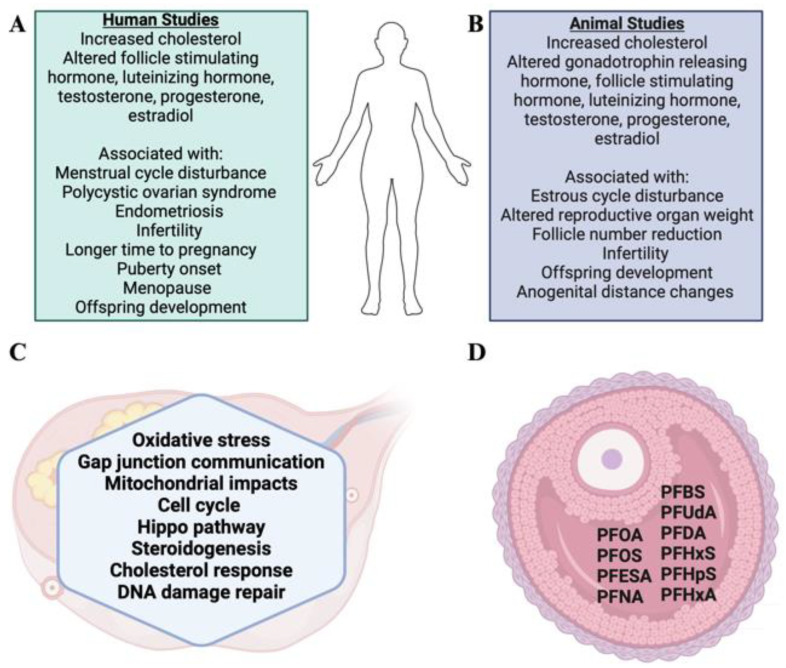
Summary of PFAS reproductive impacts. Results from (**A**) human and (**B**) animal studies related to PFAS-induced reproductive toxicity. (**C**) Molecular pathways altered in the ovary by PFAS exposure and (**D**) PFAS chemicals detected in follicular fluid from women. Created using Biorender.

Follicular fluid is a physiological and biologically relevant component found in the antral cavity of the follicle, which contains proteins, steroid hormones, polysaccharides, metabolites, reactive oxygen species, and antioxidants [149]. Follicular vascularity permits the partitioning of xenobiotics to this biofluid with close proximity to the oocyte [150]. Since the blood–follicle barrier can be crossed by albumin [151,152], it has been suggested that PFAS can be present in growing follicles [8], and indeed, PFAS have been detected in follicular fluid collected from women (Figure 1D) [125,153,154,155,156]. PFAS have a reported high blood–follicle transfer efficiency [156], and the ratio of PFAS concentration in both the serum and the FF has been positively correlated [156]. PFOA, PFOS, PFESA, PFNA, PFUndA, PFDA, PFHxS, and PFHpS were detected in FF from Chinese women [157], PFOA, PFOS, PFNA, PFUnDA, and PFDA were detected in women from Estonia and Sweden [158], and both PFOS and PFUnDA in FF were associated with dietary consumption [159]. In a small cohort of US women, PFOA, PFOS, and PFHxS were also detectable in FF [160]. Levels of PFOS in FF have been determined to be higher in women with irregular menses [125]. PFOA in FF was found to be significantly associated with elevated odds of PCOS with adjustment for confounding influences [161]. High FF levels of PFOA have been linked with a diminished ovarian reserve, suggesting that PFOA may affect the ovarian reserve function by altering the FF metabolic composition [162]. Moreover, another study determined that IVF patients who had PFAS in their FF had lower fertilization rates and a decreased number of embryos for transfer [163]. These studies suggest that the presence of PFAS in ovarian FF might lead to fertility issues.

### 4.7. Other Reproductive Pathology

As with other reproductive endpoints, variations in etiological responsibility of PFAS with endometriosis and PCOS are reported. Exposure to PFOA, PFNA, PFBS, and PFOS has been associated with endometriosis [164,165,166], although in Swedish women between 20–50 years, this was not observed [167]. Another relatively common female reproductive pathology, PCOS, is linked with PFAS exposure, including PFHxS, PFOA, and PFOS [125,167,168]. In Chinese women, PCOS-related infertility was positively associated with the PFDoA plasma level but, conversely, inversely correlated with plasma PFUnA [169], and as noted above, PFOA in FF was linked with higher odds of PCOS [161].

A clear link between ovarian cancer and PFAS exposure is not established, likely due to being understudied, with inconsistent results reported to date. Lack of association between PFOA serum concentrations and risk for ovarian cancer has been reported in one study [170], while another positively associated ovarian cancer with high PFOA serum levels [171]. In the human ovarian cancer cell lines, OVCAR-3 and Caov-3 exposed to PFOA, perfluoroheptanoic acid (PFHpA), and perfluoropentanoic acid (PFPA) and treated with carboplatin, a chemotherapeutic agent, exposure to PFAS chemicals either singly or as mixtures, increased the survival of ovarian cancer cells receiving carboplatin treatment suggesting that PFAS chemicals conferred chemotherapeutic treatment resistance on the cancer cells [172].

## 5. Developmental Effects

PFAS chemicals have been suggested to cross the placental barrier [134] and are detectable in umbilical cord blood [173,174,175,176,177]. Contradictory reports of PFAS exposure and developmental outcomes are documented. Investigations of serum levels of PFOA and PFOS and pregnancy outcomes did not find an association between miscarriage, preterm birth, and birth weight [178]. Weak associations between preeclampsia and PFOS and PFOA, as well as offspring birth defects, have been reported [178]. Maternal serum PFAS concentrations were not associated with changes in offspring birth weight [179]. Moreover, the correlation between PFOS maternal and blood cord concentrations and offspring birth weight and sex was not supported [180] in one investigation, but a weak and variable association between offspring weight, length and head circumference at birth, and maternal serum PFAS concentration was reported in another [181]. In umbilical cord blood, elevated PFOA was weakly associated with low offspring birth weight, while increased PFOS levels were linked with preterm birth [173]. Reduced birth length was reported in female fetuses due to maternal PFOS, PFNA, PFDA, PFUnA, and PFDoA [174]. The negative association between cord blood PFOS and PFOA with birth weight, ponderal index, and head circumference has been noted, but no association was determined for gestational age and newborn length [182]. Likewise, another study concluded that developmental exposure to PFOA reduces fetal growth [183], and high concentrations of PFOS and PFOA in drinking water supplies have been correlated with lower mean birth weight, preterm birth, and reduced fertility [184]. In pregnant women from San Francisco, PFAS levels and birth weight or gestational age were not determined to be linked [185]. In a different cohort, PFHxS levels and decreased birth weight were linked; however, PFUnA was associated with increased birth weight [186]. Reduced birth weight was also reported as a consequence of maternal PFNA, PFDA, PFUnA, PFDeA, and PFDoA exposure [187,188]. Anogenital distance (AGD) is a measure of endocrine disruption during *in utero* development, and a link between increased female neonatal AGD and maternal PFAS concentrations has been reported [189]. However, similar to other reproductive endpoints, shortened AGD in female infants at three months of age is associated with maternal serum concentrations of PFOS, PFHxS, and PFNA [190], while two more studies did detect a correlation between maternal serum PFAS and AGD in females [191,192].

## 6. Effect of PFAS on Female Reproduction as Determined by Studies in Animal Models

### 6.1. Reproductive Organ Weight

Several animal models have been used to try to bridge gaps in epidemiological studies regarding PFAS exposure and female reproduction and the mechanisms of action of reproductive and developmental toxicity, as summarized in Table 2. Most studies have been performed in rodent animal models; however, others have used differing animal models, including swine, cattle, and fish. In addition to humans, studies in animal models mostly focus on the legacy PFAS, and some of the results show discrepancies. Changes in reproductive organ weight have been reported in rodents, but the results are inconsistent. In lean mice exposed to 2.5 mg/kg per body weight of PFOA for 15 d, a reduction in ovarian weight was observed but not in obese mice [193]. There were no changes in uterine weight due to PFOA exposure [193]. In contrast, in prepubertal mice exposed to 0.01 mg/kg PFOA from PND 18–20, increased absolute and relative uterine weight was observed [194]. Exposure to 50 mg/kg/day PFHxS for 42 days decreased ovarian weight in mice [195]. In contrast, neonatal (PND 1–5) and juvenile (PND 26–30) female rats exposed to 1 mg/kg and 10 mg/kg PFOA, respectively, had increased ovarian weight [196]. In pregnant Kunming mice, PFOA exposure did not alter ovarian weight [197]. Similarly, mice exposed to 1, 5, 10, or 20 mg/kg of PFOA did not have alterations in ovarian or uteri weight [198], nor did adult female mice treated with 0.1 mg/kg/day of PFOS for 4 months [199]. Additionally, prepubertal female rats treated with 0.5, 1.5, and 3 mg/kg/day of PFDoA for 28 d did not have changes to ovarian or uterine weight [200]. Conversely, PFOS exposure in female zebrafish inhibited ovarian growth [201]. Other studies found different results in pathological lesions in the female reproductive tract of animals exposed to PFAS. Tubular hyperplasia in the ovaries was increased in female rats after being fed for 2 years with 1.5 mg/kg/day of ammonium perfluorooctanoate (APFO); however, a subsequent analysis did not determine any association with ovarian hyperplasia [202]. Prepubertal CD-1 mice exposed to PFOA had histopathological changes in the uterus, cervix, and vagina [194], but female Sprague Dawley rats fed with 1.3–1.8 mg/kg/day PFOS for 4 or 14 weeks did not experience histological changes in the reproductive tract [203]. Similarly, prepubertal female rats treated with PFDoA did not experience observable histomorphological ovarian or uterine changes [200], nor was any alteration to reproductive organs noted in six-week-old female rats exposed to PFBA [204].

### 6.2. Endocrine Disruption

Hormone level changes, differences in the estrous cycle, and the number of follicles have also been evaluated in different animal models to try to understand PFAS toxicity, and some inconsistencies have been observed in these studies. C57BL/6 female mice exposed to 5 mg/kg PFOA at three weeks of age for five days per week for four weeks did have altered serum E_2_; however, P_4_ serum levels were increased during the estrus and proestrus stages of the estrous cycle [205]. Exposure to 2.5 mg/kg/day PFOA for 15 d did not impact E_2_ or P_4_ serum levels; however, when samples lower than the level of detection were omitted from the E_2_ assay, E_2_ serum levels were higher in obese mice due to PFOA exposure [193]. In another study, PFOA exposure decreased serum P_4_ in pregnant mice [197]. In mice exposed to 1, 5, 10, or 20 mg/kg of PFOA, there was no impact on E_2_, but P_4_ and pregnenolone levels were decreased at 5 mg/kg exposure, and 1 mg/kg increased testosterone levels [198]. An *in vitro* study in mouse ovaries exposed to 100 µg/mL PFOA reported a decrease in E_2_ and estrone levels [198]. Secretion of P_4_ was not altered by 0.012–24 mM PFOA exposure in cultured porcine theca cells [206]. However, in granulosa cells, P_4_ and E_2_ secretion were decreased at 0.12 mM and 0.012 mM PFOA, respectively, indicating a concentration-dependent effect [206]. Adult female mice treated with 0.1 mg/kg/day of PFOS for four months had decreased serum levels of E_2_ and P_4_ at the proestrus and diestrus stages of the estrous cycle [199]. In addition, decreased LH, FSH, and gonadotropin—releasing hormone levels were also observed [199]. In prepubertal rats, PFDoA decreased serum E_2_ level at an exposure level of 3 mg/kg/day [200]. In contrast, PFOA exposure increased E_2_ and P_4_ levels in swine granulosa cells [207]. Thus, there are variations in the endocrine effects; however, disruption to hormonal homeostasis by PFAS chemicals is supported.

### 6.3. Estrous Cyclicity

In prepubertal rats, PFDoA did not induce irregularities in estrous cyclicity or timing of vaginal opening [200]. However, early vaginal opening was observed with 10 mg/kg of PFOA and 1 and 10 mg/kg of PFOS after neonatal and juvenile exposure [196]. Furthermore, the same study showed that PFOS and PFOA exposure induced irregular estrous cyclicity [196] and 1 or 10 mg/kg of PFOS for 14 d increased time spent in diestrus [208]. In contrast, PFOS did not alter the estrous cycle in rats [209] nor in mice dosed with 2.5 mg/kg of PFOA for 15 d [193] or rats exposed to PFHxS, PFUnA, and PFHxA [210,211,212]. However, mice chronically exposed to PFHxS had increased estrous cycle length with longer duration spent in diestrus [195]. Similarly, longer estrous cycles and decreased ovulation rates after exposure to PFHxS were noted in mice [213]. While no alterations in estrous cyclicity were observed in female rats dosed with 2.5 mg/kg/day of PFDoA for 14 d, exposure for 42 d caused continuous diestrus [214]. Prolonged diestrus is indictive of ovarian failure; thus, it could be reflective of entry into premature cyclicity cessation.

### 6.4. Follicular Effects

Differences in ovarian follicle number due to PFAS exposure have been reported in several studies.

In mice, PFOS exposure decreased the number of mature follicles and CL and increased the number of atretic follicles [199]. In vivo exposure to 5 mg/kg PFOA decreased the number of primordial follicles, while the number of preantral and antral follicles was increased [198]. Neonatal PFOA and PFOS exposure in rats decreased the number of secondary follicles, growing follicles, atretic follicles, and CL [196]. PFHxS exposure also decreased secondary and antral follicles and CL in mouse ovaries [190]. In pregnant mice, PFOA decreased the number of CL [197]. In vitro exposure to 50 µM of PFOA in mice increased the number of secondary follicles [215], while 100 µg/mL PFOA decreased antral follicle growth [198]. In fathead minnow, PFOS exposure decreased the number of CL and increased atretic follicles [216]. However, in contrast, rats exposed to PFDoA did not change their follicle or CL number [200,214]. Thus, follicle loss is supported as an outcome of PFAS exposures, though inconsistent findings are noted.

### 6.5. Developmental Effects

Exposure to PFAS has been shown to alter developmental outcomes. PFOA exposure increased the number of resorbed embryos at 10 mg/kg/day on gestational day (GD) 13 and increased serum E_2_ on GD 7 in mice [197]. Furthermore, pregnant CD-1 mice exposed to PFOA had decreased fetal and placental weight, an increased number of resorptions and dead fetuses, and a decreased live fetus number in a dose-dependent manner [217]. Similarly, PFOS exposure in mice decreased maternal body weight gain and fetal and placental weight dose-dependently [218]. Mice exposed to PFOA had early pregnancy loss, compromised postnatal survival, and delayed growth and development [219]. Exposure to PFOS in rats from GD 0 until PND 20 did not cause alterations in the number of litters, gestation length, number of implantation sites, and resorptions [220]. Moreover, *in utero* exposure to PFOS in mice and rats compromised postnatal survival and delayed growth and development [221]. In addition, exposure to PFOS in swordtail fish caused female reproductive and developmental toxicity [222]. Bovine oocytes exposed to PFNA *in vitro* during maturation and then fertilized had impaired developmental competence during maturation and alterations in lipid accumulation in the blastocysts [223]. Pregnant female rats dosed with 2.5 mg/kg/day PFDoA either died or were moribund at the end of the pregnancy with signs of hemorrhage in the implantation sites and congestion of the endometrium, and only one female delivered pups that had low body weight [214]. In contrast to these studies in which developmental impacts of PFAS were reported, several others did not observe impacts. Sprague Dawley rats exposed to PFHxS did not have reproductive or developmental effects in either the dams or the offspring [210]. In CD-1 mice, PFHxS did not impact postnatal survival, development, and vaginal opening in F_1_ mice; however, the live litter size was decreased [224]. In rodents, exposure to GenX caused placental abnormalities, reduced pup birth weight, and increased neonatal mortality [225,226]. In pregnant female rats, exposure to PFUnA did not change the sex ratio of live pups; however, the body weight of pups was decreased on PND 0 and 4 [212].

Some multi-generational studies with PFAS have been reported. In a two-generation study, females exposed to APFO 70 d before mating did not affect estrous cyclicity, fertility, pregnancy, the natural length of gestation, or the number of litters [227]. In the F_1_ generation, there were no effects on female reproduction, but a delay in vaginal opening was noted [227]. Exposure to PFHxA did not affect mating, fertility, gestation length, number of implantation sites, litter size, sex ratio, or pup survival in F_0_ and F_1_ generations [50,211]. There were no fertility or reproductive effects, including infertility, estrous cyclicity, pregnancy, mating, and natural delivery after exposure to PFBS in a two-generation study in rats [228]. In zebrafish F_1_ and F_2_ generations, PFOS exposure caused deformities and other developmental outcomes [229,230]. Additionally, F_1_ embryos exposed *in utero* to PFOS had malformations and increased mortality [201]. Furthermore, zebrafish exposure to F53B delayed hatchings, increased birth defects, and reduced survival rates [231]. However, developmental toxicity was not observed in zebrafish embryos exposed to GenX and ADONA [232].

Taken together, studies in animal models indicate that PFAS exposures cause female reproductive toxicity by inducing changes in reproductive organs, endocrine disruption, alterations in the estrous cycle, differences in the number of follicles and CL, and developmental toxicity. However, these findings have some inconsistencies, and the time and route of exposure, type of PFAS chosen, type of animal model chosen, and developmental status of the animal are likely contributors to the variation in endpoint impacts noted.

## 7. Possible Mechanisms of Action by which PFAS Exposure Causes Female Reproductive Toxicity

A lack of understanding of the mechanisms of action in which PFAS causes reproductive toxicity exists. However, several studies have evaluated molecular endpoints to generate an understanding of modes of action related to female reproductive toxicity, as summarized below and in Figure 1C. PFAS can interact with estrogen receptors [200,233,234,235,236] and are endocrine disruptors since they can alter hormone levels. Reproductive toxicity is suggested to be increased as the chemical carbon chain length increases and a sulfonate group is added [237]. Additionally, exposure to PFOA and PFHxS disrupts gap junction intercellular communication in cumulus cell–oocyte complexes [213,238], which could lead to alterations in the growth and development of the oocyte [239]. Exposure to PFOS and PFHxS increased the intracellular level of reactive oxygen species in mouse oocytes [237]. Additionally, in pregnant and non-pregnant mouse ovaries, oxidative stress and apoptosis were observed after exposure to PFOA [197,238]. Further, in swine granulosa cells, PFOA exposure induced cell viability but inhibited free radical production, altering normal ovarian homeostasis [240]. Additionally, PFOS and PFHxS induced mitochondrial depolarization, chromosome misalignment, abnormal assembly of F-actin and spindle, and compromised developmental competence of oocytes [237]. Moreover, in mice, PFOA exposure altered abundance genes and proteins with roles in the cell cycle, Hippo pathway, steroidogenesis, DNA damage sensing and repair, and reproduction [193,198,215]. PFOS also altered the mRNA abundance of genes involved in estrogen receptor function, early thyroid development, and steroidogenic enzyme synthesis in zebrafish embryos [241]. Related further to steroidogenesis, PFDoA affected ovarian levels of genes involved in steroidogenesis and cholesterol transport [200]. In juvenile Atlantic cod fish ovarian tissue, exposure to PFOS, PFOA, and PFNA changed levels of genes involved in cellular signaling, adhesion, cytoskeleton, remodeling, lipid metabolism, ovarian development, steroidogenesis, cancer, and apoptotic and proapoptotic reproduction signaling pathways [242]. Alterations in the levels of the genes and proteins involved in the biological and molecular functions mentioned could lead to alterations in ovarian homeostasis, leading to reproductive toxicity.

Another possible mechanism that can lead to female reproductive toxicity is through the activation of peroxisome proliferator-activated receptor (PPARs) signaling pathways (reviewed in Ding et al., 2020 [8]). The PPAR isoforms α, β/δ, and γ are transcription factors that are ligand-specific and have different functions. PPARs are present in the ovary and involved in different processes (reviewed in Komar, 2005 [243]), including cell cycle, steroidogenesis, apoptosis, angiogenesis, lipid metabolism, and tissue remodeling [243,244,245]. PPARα is located primarily in theca cells and stroma, PPARβ/δ has a widespread ovarian location, and PPARγ is present in the oocyte, theca, and granulosa cells of different species [243]. PFAS can interact with PPAR isoforms [246] in different tissues, including the ovary, possibly leading to alterations in ovarian function and other female reproductive effects [8], though this is not well explored.

Cholesterol is a precursor of bile acids, steroid hormones, and cholesterol esters, and the cholesterol metabolite, cholesta-3,5-diene, regulates cholesterol biosynthesis and absorption and acts on transcription factors and receptors [247]. In both men and women, positive associations between PFAS exposure and increased serum cholesterol have been reported [248,249,250,251,252,253,254]. Both cholesterol and cholesta-3,5-diene are increased by PFOA exposure in female mice [255], and a PFAS-induced increase in cholesterol has been noted in other animal investigations [252,253,256]. In male and female C57BL/6J mice exposed to PFAS via drinking water, increased circulating cholesterol was observed [256] and was also recapitulated in female C57BL/6 mice exposed to PFOA [257]. The abundance of ovarian cholesterol-responsive proteins (RALY, CFTR, LRP1, NAXE, APOA4, APOA2, SOAT1, EHD1, HMGCS2, and CES1C) was altered in PFOA-exposed mice [255], suggesting that the ovary is responsive to systemic cholesterol fluctuations. Since cholesterol is a precursor of steroid hormones and cholesterol excess has been implicated in aberrant ovarian function [258], PFAS exposure could impact fertility through altering steroidogenesis, concomitant with endocrine disrupting effects of PFAS reported in human and animal studies.

## 8. Conclusions

There is support in the current literature for PFAS chemicals being considered female reproductive toxicants with effects on the endocrine system, folliculogenesis, puberty and menopause timing, fertility, pregnancy success, and development. Effects including altered cholesterol levels, menstrual/estrous cycle disturbance, offspring developmental perturbations, and infertility are noted both in human epidemiological and animal studies, as summarized in Figure 1. There are inconsistencies across studies, likely attributable to developmental status, geographical location, and exposure paradigm (chemical, dose, duration, and route).

## Figures and Tables

**Table 1:** PFAS half-life of elimination in different species.

Species														
PFAS	Humans		Rat		Mouse		Non-Human Primates		Pigs		Cattle		Chicken	
	F	M	F	M	F	M	F	M	F	M	F	M	F	M
PFOS	3.4–5.4 y [32,33]		100 d [39]		30.4–37.8 d [40]	36.4–42.8 d [40]	110–200 d [40]	131–200 d [40]	1.7 y [27]		38.7–106 d [54,55]	120 d [55]		125 d [57]
			62–71 d [40]	38–41 d [40]									* 3.5 d [29]	
PFOA	2.7–3.8 y [32,33]		2–4 h [26,39]	4–9 d [26,39]	16 d [50]	22 d [50]	32.6 d [52]	20–21d [52]	236 d [27]		1.3 d [54]	~19.2 h [56]		5.2 d [57]
													* 5.4 d [58]	
PFNA	2.5–4.3 y [34]		1.4–2.44 d [41,42,43]	29.5–47 d [41,42,43]	25.8–68.4 d [41]	34.3–68.9 d [41]					8.7 d [54]			
PFDA	4.5–12 y [34]		58.6 d [42]	39.9 d [42]							19 d [54]			
PFHxS	5.3–8.5 y [32,33]		1.12 h–1.7 d [45,46]	215.9–29 d [43,45,46]	24.9–26.8 d [45]	27.9–30.5 d [45]	87 d [45]	141 d [45]	1.9 y [27]				* 7 d [58]	
PFBA	~3 d [35]		1.03–1.76 h [35]	6.38–9.22 h [35]	2.79–3.08 h [35]	5.22–16.25 h [35]	40.3 h [35]	41.0 h [35]						
PFHxA	32 d [36]		0.42–3 h [47,48]	1–3 h [47,48]	1 h [36]		2.4 h [47]	5.3 h [47]	4.1 d [27]					
PFBS	28 d [37]		0.64–4 h [37,46]	2.1–4.5 h [37,46]	4.5 h [50]	5.8 h [50]	8 h–3.5 d [37,47]	15 h–4 d [37,47]	43 d [27]					
GenX			8 h [49]	3 h [49]	18 h [49]	21 h [49]								
F53B	15.3 y [38]													

Abbreviations: PFAS—per- and polyfluoroalkylated substances; PFOS—perfluooroctane sulfonic acid; PFOA—perfluorooctanoic acid; PFNA—perfluorononanoic acid; PFDA—perfluorodecanoic acid; PFHxS—perfluorohexane sulfonic acid; PFBA—perfluorobutanoic acid; PFHxA—perfluorohexanoic acid; PFBS—perfluorobutanesulfonic acid; GenX—hexafluoropropylene oxide dimer acid and ammonium salts; F53B—chlorinated polyfluoroalkyl ether sulfonate; y = year; d = days; h = hours; * in eggs. Superscripts indicate citation in which data published.

**Table 2:** Summary of effect of PFAS on female reproduction as determined by studies in animal models [205,206,207,208,209,210,211,212,213,214,215,216,217,218,219,220,221,222,223,224,225,226,227,228,229,230,231,232].

PFAS Substance	Species and Strain	Dose	Exposure Route	Duration of Exposure	Findings	Reference
PFOA	KK.Cg-a/a mice	2.5 mg/kg	Oral	15 d	Reduction in ovarian weight No changes to E_2_ and P_4_ serum levels No alterations to estrous cycle	[193]
PFOA	CD-1 mice	0.01 mg/kg	Gavage	PND 18–20	Increased absolute and relative uterine weight Histopathological changes in the uterus, cervix and vagina	[194]
PFOA	Sprague-Dawley rats	0.1, 1, 10 mg/kg	Injected	PND 1–5 or PND 26–30	Increased ovarian weight Increased E_2_ and LH levels Irregular estrous cyclicity Early vaginal opening Decreased number of secondary follicles and growing follicles	[196]
PFOA	Kunming mice	2.5, 5, 10 mg/kg/d	Gavage	GD 1–7 or GD 1–13	No alterations in ovarian weight Increased E_2_ serum levels at GD 7 Decreased P_4_ serum levels at GD 13 Reduced number and size of CL Increased number of resorbed embryos on GD 13	[197]
PFOA	CD-1 mice	1, 5, 10, 20 mg/kg	Oral	10 d	No alterations in ovarian or uterine weight No alterations in E_2_ levels Decreased P_4_ and pregnenolone levels Increased testosterone levels Decreased number of primordial follicles Increased number of preantral and antral follicles	[198]
	CD-1 mouse ovaries	100 µg/mL	*In vitro*	96 h	Decreased E_2_ and estrone levels Decreased antral follicle growth	
PFOA	C57BL/6	5 mg/kg	Gavage	5 d per w for 4 w	No alterations in E_2_ serum levels Increased P_4_ serum levels	[205]
PFOA	Porcine theca cells	0.0012 mM	*In vitro*	24 h	No alterations on P_4_ levels	[206]
	Porcine granulosa cells	0.12, 0.012 mM	*In vitro*	24 h	Decreased E_2_ and P_4_ levels	
PFOA	Swine granulosa cells	2, 20, 200 ng/mL	*In vitro*	48 h	Increased E_2_ levels Alterations in levels of P_4_	[207]
PFOA	CD-1 mice	50 µM	*In vitro*	96 h	Increased number of secondary follicles	[215]
PFOA	CD-1 mice	2, 10, 25 mg/kg/d	Gavage	GD 11–16	Decreased fetal and placental weight Increased number of resorptions and dead fetuses Decreased live fetus number	[217]
PFOA	CD-1 mice	1, 3, 5, 10, 20, 40 mg/kg/d	Gavage	GD 1–17	Early pregnancy loss Compromised postnatal survival Delayed growth and development	[219]
PFOA	CD-1 mice	1, 5 mg/kg/d	Gavage	ED 1.5–11.5 or 17.5	Placental abnormalities Reduced embryo growth	[225]
APFO	Sprague-Dawley rats	1.5, 15 mg/kg/d	Oral	2 y	Tubular hyperplasia in the ovaries	[202]
APFO	Sprague-Dawley rats	1, 3, 10, 30 mg/kg	Oral	>70 d	No effects to estrous cyclicity, fertility, pregnancy, natural length of gestation on F_0_ Delayed vaginal opening on F_1_ generation	[227]
PFOS	Sprague-Dawley rats	0.1, 1, 10 mg/kg	Injected	PND 1–5 or PND 26–30	Increased E_2_ and LH levels Irregular estrous cyclicity Early vaginal opening Decreased number of secondary follicles, growing follicles, atretic follicles and CL	[196]
PFOS	ICR mice	0.1 mg/kg/d	Gavage	4 m	No alterations in ovarian weight Decreased serum levels of E_2_ and P_4_, Alterations in LH, FSH, and GnRH level Decreased number of mature follicles and CL Increased number of atretic follicles	[199]
PFOS	Sprague-Dawley rats	1.3–1.8 mg/kg/d	Oral	4 or 14 w	No alterations to uterus, cervix or vagina	[203]
PFOS	Zebrafish	50, 250 µgL^−1^	Via tank water	70 d	Inhibited ovarian growth Increased malformations and mortality	[201]
PFOS	Sprague-Dawley rats	1, 10 mg/kg	Intraperitoneal injection	14 d	Irregular estrous cyclicity	[208]
PFOS	Crl:CD^®^ (SD)IGS BR VAF^®^ rats	0.1, 0.4, 1.6, 3.2 mg/kg/d	Gavage	6 w	No alterations in estrous cyclicity	[209]
PFOS	Fathead minnow	0.3, 1 mg/L	Via tank water	21 d	Decreased number of CL Increased number of atretic follicles	[216]
PFOS	CD-1 mice	0.5, 2, 8 ng/kg/d	Gavage	GD 11–16	Decreased maternal body weight gain, fetal and placental weight	[218]
PFOS	Sprague-Dawley rats	0.1, 0.3, 1.0 mg/kg/d	Gavage	GD 0–20	No alterations in the number of litters, gestation length, number of implantation sites, and resorptions	[220]
PFOS	CD-1 mice	1, 5, 10, 15, 20 mg/kg/d	*In utero*	GD 1–18	Compromised postnatal survival Delayed growth and development	[221]
	Sprague-Dawley rats	1, 2, 3, 5, 10 mg/kg/d		GD 2–21		
PFOS	Swordtail fish	0.1, 0.5, 2.5 mg/L	Via tank water	6 w	Female reproductive and developmental toxicity	[222]
PFOS	Zebrafish	5, 50, 250 µg/L	Via tank water	5 m	Alterations to embryonic growth, reproduction and offspring development	[229]
PFOS	Zebrafish	0.6, 100, 300 µg/L	Via tank water	0–180 dpf	Increased mortality and developmental outcomes	[230]
PFHxS	ICR mice	5, 50 mg/kg/d	Intragastric administration	42 d	Decreased ovarian weight Prolonged estrous cyclicity Decreased number of secondary follicles, antral follicles, and CL	[195]
PFHxS	Crl:CD^®^ (SD)IGS BR VAF/Plus ^®^ rats	0.3, 1.3, 10 mg/kg/d	Gavage *In utero*	14 d prior to cohabitation through GD 21, GD 25, or PND 22	No alterations to estrous cyclicity No reproductive or developmental effects	[210]
PFHxS	CD-1 mice	25.1, 62.5 mg/kg	Intraperitoneal injection	Single dose	Irregular estrous cyclicity Decreased ovulation rate	[213]
PFHxS	Crl:CD1(ICR) mice	0.3, 1, 3 mg/kg/d	Gavage, *in utero*, via lactation	42 d 14 d	Decreased litter size No alterations in postnatal survival, development, and vaginal opening	[224]
PFDoA	Sprague- Dawley rats	0.5, 1.5, 3 mg/kg/d	Oral	28 d	No changes in ovarian and uterine weight No histomorphological ovarian or uterine changes Decreased E_2_ levels No alterations in estrous cyclicity No alterations in vaginal opening No alterations to follicle and CL numbers	[200]
PFDoA	Crl:CD (SD) rats	2.5 mg/kg/d	Gavage	42 d	Irregular estrous cyclicity No changes in CL numbers Maternal mortality Stillbirths Developmental toxicity	[214]
PFBA	Sprague-Dawley rats	1.2, 6, 30, 150 mg/kg/d	Gavage	28 d or 90 d	No changes in ovarian and uterine weight	[204]
PFBS	Sprague-Dawley rats	30, 100, 300, 1000 mg/kg/d	Gavage	>70 d	No fertility or reproductive effects to dams and female offspring	[228]
PFHxA	Crl:CD (SD) rats	20, 100, 500 mg/kg	Gavage	90 d 4 m GD 6–20	No alterations in estrous cyclicity No reproductive and developmental effects	[211]
PFHxA	Crl:CD (SD) rats	50, 150, 300 mg/kg	Gavage	39–52 d	No reproductive or developmental toxicity	[50]
PFUnA	Crl:CD (SD) rats	0.1, 0.3, 1.0 mg/kg/d	Gavage *In utero*	41–46 d	No alterations to estrous cyclicity Decreased body weight of pups	[212]
PFNA	Bovine oocytes	0.1, 10 mg/mL	*In vitro*	22 h	Impaired oocyte developmental competence Alterations to lipid accumulation in blastocysts	[223]
GenX	CD-1 mice	1, 5 mg/kg/d	Gavage	ED 1.5–11.5 or 17.5	Placental abnormalities Reduced embryo growth	[225]
GenX	Sprague-Dawley rats	1, 3, 10, 30, 62.5, 125, 250 mg/kg/d	Gavage	GD 17–21 or GD8-PND 2	Decreased pup weight Increased neonatal mortality	[226]
GenX	Zebrafish	0.04, 0.1, 0.4, 1.1, 3.1, 9.3, 27.2, 80.0 µM	Filter inserts containing zebrafish embryos in 96-well culture trays with DMSO or DI water	0–5 dpf	No developmental toxicity	[232]
ADONA						
F53B	Zebrafish	1.5, 3, 6, 112 mg/L	Via tank water	6–132 hpf	Delayed hatchings Increased birth defects Reduced survival rates	[231]

Abbreviations: PFAS—per- and polyfluoroalkylated substances; PFOS—perfluooroctane sulfonic acid; PFOA—perfluorooctanoic acid; PFNA—perfluorononanoic acid; PFHxS—perfluorohexane sulfonic acid; PFBA—perfluorobutanoic acid; PFHxA—perfluorohexanoic acid; PFBS—perfluorobutanesulfonic acid; PFUnA—perfluoroundecanoic acid; PFDoA—perfluorododecanoic acid; GenX—hexafluoropropylene oxide dimer acid and ammonium salts; F53B—chlorinated polyfluoroalkyl ether sulfonate; APFO—ammonium perfluorooctanoate; ADONA—3H-4,8-dioxanonanoate; y = year; m = months; w = weeks; d = days; h = hours; GD = gestational day; PND = postnatal day; ED = embryonic day; dpf = days post-fertilization; hpf = hours post-fertilization.

## Data Availability

Not applicable.
